# Harnessing the potential of HLA-G in cancer therapy: advances, challenges, and prospects

**DOI:** 10.1186/s12967-024-04938-w

**Published:** 2024-02-03

**Authors:** Siyuan Wang, Jiaxin Wang, Yu Xia, Le Zhang, Yueqiang Jiang, Man Liu, Qinglei Gao, Cuntai Zhang

**Affiliations:** 1 Department of Geriatrics, Tongji Hospital, Tongji Medical College, Huazhong University of Science and Technology, 1095 Jiefang Avenue, Wuhan, 430030 China; 2 Key Laboratory of Vascular Aging, Ministry of Education, Tongji Hospital, Tongji Medical College, Huazhong University of Science and Technology, 1095 Jiefang Avenue, Wuhan, 430030 China; 3 Department of Urology, Tongji Hospital, Tongji Medical College, Huazhong University of Science and Technology, 1095 Jiefang Avenue, Wuhan, 430030 China; 4 Cancer Biology Research Center (Key Laboratory of Chinese Ministry of Education), Tongji Hospital, Tongji Medical College, Huazhong University of Science and Technology, 1095 Jiefang Ave, Wuhan, 430030 China; 5 Department of Gynecology and Obstetrics, Tongji Hospital, Tongji Medical College, Huazhong University of Science and Technology, 1095 Jiefang Avenue, Wuhan, 430030 China

**Keywords:** HLA-G, Cancer, Immune checkpoint blockades, Immunotherapy

## Abstract

Immune checkpoint blockades have been prized in circumventing and ablating the impediments posed by immunosuppressive receptors, reaching an exciting juncture to be an innovator in anticancer therapy beyond traditional therapeutics. Thus far, approved immune checkpoint blockades have principally targeted PD-1/PD-L1 and CTLA-4 with exciting success in a plethora of tumors and yet are still trapped in dilemmas of limited response rates and adverse effects. Hence, unveiling new immunotherapeutic targets has aroused immense scientific interest in the hope of expanding the clinical application of immune checkpoint blockades to scale new heights. Human leukocyte antigen-G (HLA-G), a non-classical major histocompatibility complex (MHC) class I molecule, is enriched on various malignant cells and is involved in the hindrance of immune effector cells and the facilitation of immunosuppressive cells. HLA-G stands out as a crucial next-generation immune checkpoint showing great promise for the benefit of cancer patients. Here, we provide an overview of the current understanding of the expression pattern and immunological functions of HLA-G, as well as its interaction with well-characterized immune checkpoints. Since HLA-G can be shed from the cell surface or released by various cells as free soluble HLA-G (sHLA-G) or as part of extracellular vesicles (EVs), namely HLA-G-bearing EVs (HLA-G_EV_), we discuss the potential of sHLA-G and HLA-G_EV_ as predictive biomarkers. This review also addresses the advancement of HLA-G-based therapies in preclinical and clinical settings, with a focus on their clinical application in cancer.

## Background

In the process of oncogenesis and development, malignant tumor cells evolve constantly and strive hard to build strong defenses against the immune system [1]. Tumor cells take full advantage of immune checkpoint molecules to restrain the function of immune cells, protecting themselves from attacks and achieving immune escape. On the contrary, a growing body of evidence demonstrates that strategies targeting immune checkpoints exhibit the potential to restore or enhance the anti-tumor capacity [2], leading to the emergence of immune checkpoint blockade (ICB) therapy. To date, several immune checkpoint targeting agents have gained approvals for the treatment of multiple cancers as monotherapy or in combination with other therapeutics [3–6]. With the rise of ICB in immuno-oncology, the treatment of oncology has also entered the era of immunotherapy. Despite the unprecedented breakthrough achieved by ICB therapies, considerable challenges persist, namely the limited response rate, severe hazardous adverse events, and acquired resistance [7]. More than half of these patients are likely to experience immune-related adverse events (irAEs), with 0.3 ~ 1.3% of the cases being fatal, leading to treatment interruption and poor outcomes [8]. Therefore, identification of novel immune checkpoints and formulation of innovative strategies are urgently needed, thereby broadening the clinical application spectrum of ICB therapies.

Human leukocyte antigen-G (HLA-G), a non-classical major histocompatibility complex (MHC) class I molecule, was demonstrated to be integral to maternal–fetal tolerance initially and deemed to be a vital immune checkpoint. HLA-G engages with ILT2/4 and KIR2DL4 to elicit the suppression of immune effector cells, including cytotoxic T cells and natural killer (NK) cells, as well as the expansion of immunosuppressive cells like Treg cells and myeloid-derived suppressor cells (MDSCs), resulting in an immunosuppressive microenvironment and contributing to the immune escape of tumor cells [9]. Due to the promising potential of HLA-G blockade, ongoing researches about why and how HLA-G-based therapeutic approaches quell the malignant phenotype are increasing, as exemplified by the deciphering of the roles of HLA-G positive cancer cells in colorectal cancer using spatial and single-cell transcriptomics [10], and the employment of HLA-G-targeted CAR-T cells for the direct killing of EGFR-mutated and overexpressed oral cancers [11]. In addition, several clinical trials have been designed to evaluate the effectiveness of HLA-G inhibitors as monotherapy or in combination therapy for the treatment of solid tumors. Therefore, HLA-G-based therapeutic approaches are on the way to become a hot spot in the field of tumor immunotherapy.

In this review, we overview the fundamentals and immunobiology of HLA-G and pinpoint its discrepancy with PD-L1. In addition, we highlight the latest preclinical and clinical trial advances, with the aim of providing further insight into HLA-G-based modalities for cancer treatment and proposing new perspectives on targeted therapy for cancer patients.

### Molecular structure and distribution characteristics of HLA-G

The HLA-G gene, being highly homologous to the classical MHC molecules, is located in chromosome 6p21.3 containing eight exons encoding the signal peptide, the extracellular α1-α3 domain, the transmembrane domain, and the intracellular region, successively. The second codon of exon 6 acts as an early stop codon and the resulting intracellular cytoplasmic tail of HLA-G is shorter compared to the classical MHC molecules [9]. The HLA-G gene also comprises seven introns. Moreover, it has been well-established that at least seven different isoforms were generated via alternative splicing. Isoforms HLA-G1, HLA-G2, HLA-G3, and HLA-G4 retain the transmembrane domain to express the corresponding membrane-bound proteins, whereas the HLA-G5, HLA-G6, and HLA-G7 exist in soluble form due to the absence of transmembrane domain [12–14]. The characteristics of each isoform are summarized in Table 1.

**Table 1:** Characteristics of seven identified HLA-G isoforms

Isoform	Type	Alternative splicing	Structure characteristics
HLA-G1	Membrane-bound	Full-length, wild type	Has α1, α2, and α3 domains, non-covalently bind to β2-microglobulin (β2m)
HLA-G2	Membrane-bound	Without exon 3	Has α1 and α3 domains, lack of α2 domain, non-covalently bind to β2m
HLA-G3	Membrane-bound	Without exon 3 and exon 4	The shortest isoform, only has α1 domain
HLA-G4	membrane-bound	Without exon 4	Has α1 and α2 domains, lack of α3 domain
HLA-G5	Soluble	Contains intron 4 (with an early stop codon)	Without transmembrane domain, has α1, α2, and α3 domains, non-covalently bind to β2m
HLA-G6	Soluble	Contains intron 4 (with an early stop codon)	Without transmembrane domain, has α1 and α3 domains, non-covalently bind to β2m
HLA-G7	Soluble	Contains intron 2 (with an early stop codon)	Without transmembrane domain, only has α1 domain

Unlike classical MHC class I molecules, the expression of HLA-G is highly restricted. In physiological conditions, HLA-G is exclusively found in a few tissues and cells, including extravillous trophoblasts of the placenta [5], cornea [6], and thymus epithelial cell [7], contributing to immune tolerance through interaction with corresponding ligands on immune cells. However, overexpression of HLA-G is induced under multiple pathological conditions, including malignant tumors, viral or microbial infections [8–11], and autoimmune diseases [12, 13] Convergent data implicated that HLA-G was enriched in several tumors, thereby fueling the immune evasion.

### Underlying mechanisms regulating HLA-G expression

**Fig. 1:**
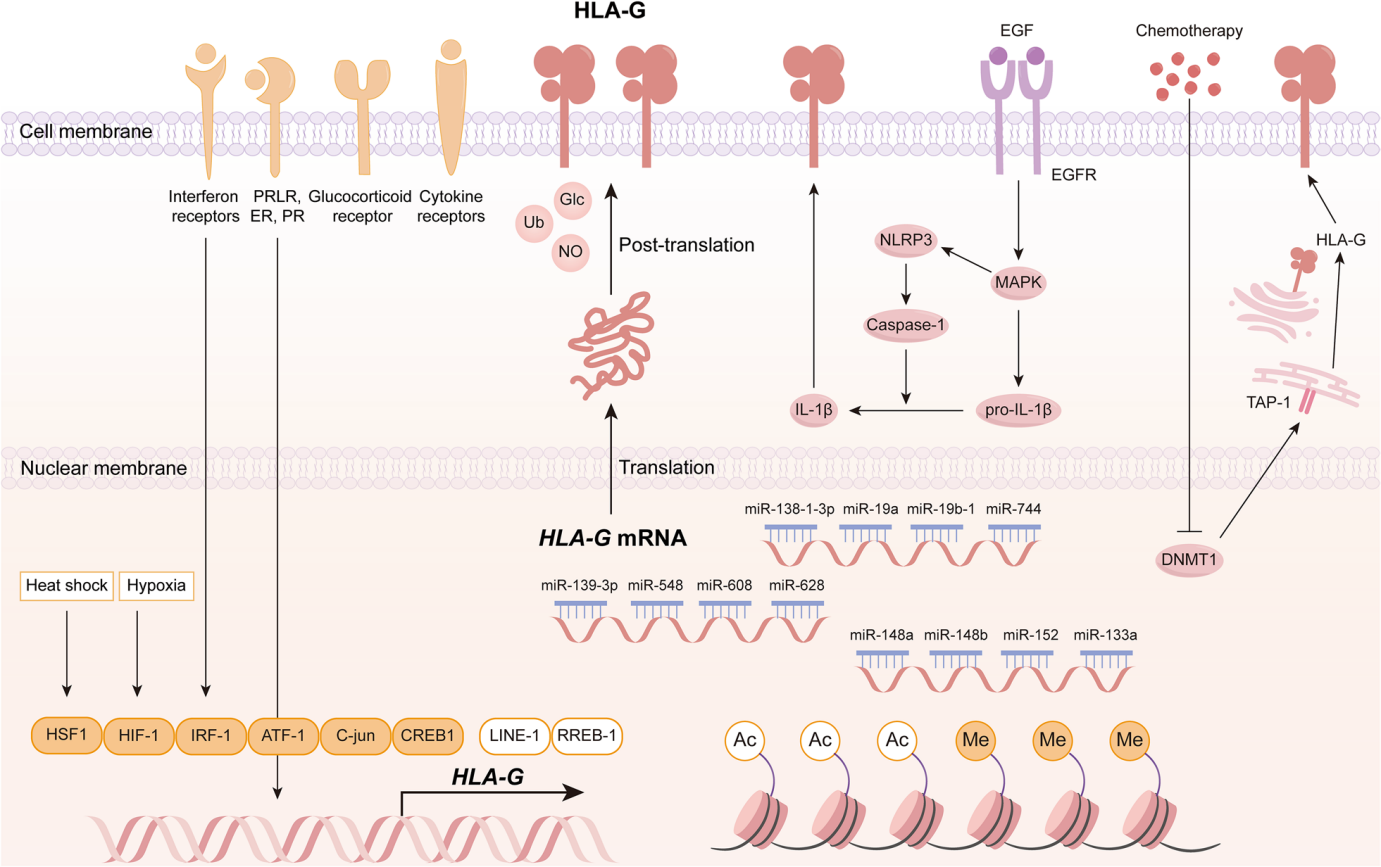
Regulation of HLA-G expression. The transcriptional activity of HLA-G is modulated by epigenetic alterations and several transcription factors in response to environmental and drug stimuli. A number of miRNAs can directly target the 3'UTR of HLA-G mRNA and impede HLA-G translation. HLA-G is also subjected to diverse post-translational modifications, including ubiquitination, nitration, and glycosylation. Moreover, various hormones, cytokines, and chemotherapy drugs can enhance HLA-G expression via direct binding with HLA-G gene or activation of downstream signaling pathways. *Ac* acetylation, *Me* methylation, *Ub* ubiquitination, *Glc* glycosylation, *NO* nitration

Several mechanisms are involved in the comprehensive modulation of HLA-G, namely epigenetic regulation, transcription factors, post-transcriptional regulation, and post-translational modification, as presented in Fig. 1.

1. Epigenetic regulation

Covalent binding of methyl group (-CH3) to cytosine residues could promote methylation of CpG islands in the HLA-G promoter region, silencing the transcription of *HLA-G* gene [15]. In this context, expression of DNA methyltransferases (DNMTs), a contributing factor to hypermethylation of promotor, was inversely correlated with HLA-G protein level [16]. On the contrary, DNMT inhibitor 5-Aza could induce a rise in *HLA-G* mRNA and protein in breast cancer cells [17], which was also confirmed in renal cell carcinoma, melanoma, pancreatic cancer, ovarian cancer, glioblastoma and choriocarcinoma [18–22]. In addition, histone acetylation was augmented in the presence of histone deacetylase inhibitor (HDACi) sodium butyrate, valproic acid, or trichostatin A (TSA), reinvigorating the transcription of *HLA-G* [19, 23]. Indeed, as one of the important intrinsic drivers of gene regulation, epigenetics could be widely exploited in the field of cancer treatment. Given that inhibitors of epigenetic regulators could potentiate HLA-G expression, their combination with HLA-G blockade deserves further exploration as novel therapeutics.

- 2. Transcriptional regulation

In addition to epigenetic mechanisms, transcriptional regulation mediated by multiple regulatory elements is involved in the regulation of HLA-G expression. It should be noted that the promoter of HLA-G is extremely divergent from that of any other MHC class I gene. The promoter regions of MHC class I genes contain regulatory modules that activate transcription, mainly including enhancer A, the interferon-stimulated responsive element (ISRE), and the SXY module. However, in HLA-G promoter, the enhancer A and SXY modules are modified, while the ISRE is absent. HLA-G exhibited a strong binding affinity to the p50 homodimeric subunit of NF-κB due to the sequence differences in the enhancer A (κB1 and κB2 sites), whereas none of the other NF-κB subunits with transcriptional properties, including p65 and c-Rel, recognized these sites [24]. Moreover, the MHC master transcription factors NLRC5 and CIITA failed to bind to the HLA-G gene promoter owing to the absence of the conserved SXY module [25]. As early as 2002, transcriptional regulators C-jun, cAMP-responsive element binding protein (CREB)-1, and ATF1 have been proven to engage with the three CRE/TRE regulatory elements in the *HLA-G* promoter region, potentiating the basal level of *HLA-G* promoter activity through trans-activation [26]. Consistently, Michael Friedrich et al. have recently validated the binding of CREB to *HLA-G* promotor sequences in renal cancer cells [17]. In addition, the binding of IRF-1, HIF-1, or HSF-1 to corresponding elements augmented HLA-G transcription in response to external stimuli, respectively [27–29]. Unlike HLA-E and HLA-F, HLA-G was barely responsive to the regulation of RFX5, due to the lack of transcriptional scaffold created by RFX5, resulting in the inability of NLRC5 and CIITA to bind to the promoter [30]. Given its unique transcriptional regulation pattern, it is reasonable to conclude that HLA-G functions distinctively throughout the immune response. Conversely, the long interspersed nuclear elements (LINE)-1 scattered throughout the upstream region of *HLA-G* leading to the silencing of the gene [31], whereas the binding of Ras responsive element binding protein (RREB)-1 to Ras responsive element was capable of restraining *HLA-G* transcription [32], which might partially account for the restricted expression of HLA-G under physiological conditions. Overall, novel transcription factors of HLA-G remain to be explored in order to screen attractive candidate drugs that might expand the applicability of HLA-G-based therapeutics.

3. Post-transcriptional regulation

MicroRNAs (miRNAs) are a class of highly conserved endogenous non-coding single-stranded RNAs with a total length of approximately 22 nt, modulating the transcription of target genes via base-pair complementation [33]. Indeed, a number of miRNAs could interact with the 3'UTR region to hinder the translation of *HLA-G*, including miR-148a, miR-148b, miR-152, miR-133a, miR-139-3p, miR-548, miR-608, miR-628, miR-138-1-3p, miR19a, miR-19b-1, miR744 [34–42]. The varying levels of HLA-G expression within the tissues might be attributed to the presence of different types and amounts of miRNAs. Placental tissues exhibited relatively low expression of miR-148a and miR-152, making the placenta one of the few tissues rich in HLA-G [38]. Conversely, the high expression of miR-148a and miR-152 has been demonstrated to be related to the reduced HLA-G protein level in renal cancer and colon cancer [34, 43]. Notably, miRNAs differed in affinity for 3'UTR sequences in HLA-G, as denoted by the fact that miR-152, miR148a, miR-148b, and miR-133a had sequentially weaker affinity for HLA-G validated by miTRAP technology [34]. In addition, miR-139-3p could bind to the non-polymorphic sequences in *HLA-G* 3'UTR region specifically, while miR-608 exclusively bound to the polymorphic sequences [37]. Convincing data is required to elucidate the underlying mechanisms of miRNAs regulation and their correlation with cancers.

4. Post-translational modification

Protein expression and function could be modified by the addition or removal of specific chemical groups to amino acids, namely the post-translational modifications, such as phosphorylation, acetylation, ubiquitination, and glycosylation, which together constitute a complex network of regulation. Ubiquitinated HLA-G complexes have been found in the circulation and unleashed as exosomes, favoring tumor spread [44]. The detection of HLA-G nitration in patient plasma and exudate might be associated with the metalloproteinase-mediated shedding of HLA-G from cell surface, and that nitrated HLA-G retained its original biological function and effectively inhibited NK cytotoxicity [45, 46]. HLA-G in body fluids could be used as a humoral biomarker for several diseases, especially malignancies. Thus, ascertaining whether the measurement of ubiquitinated or nitrated HLA-G would achieve better specificity and sensitivity will be crucial, offering predictive and therapeutic opportunities. Moreover, glycosylated HLA-G was produced by placental trophoblast cells and secreted into the amniotic fluid, being crucial for the maintenance of maternal–fetal immune tolerance [47], but mechanistic evidence for the role of HLA-G in tumor immune-evasive is still missing.

5. Others

In addition, hormones and cytokines have also been reported to modulate HLA-G expression. Progesterone could directly bind to the progesterone response element (PRE) in the HLA-G promoter and induce HLA-G transcription in trophoblast cells, breast cancer cells, and choriocarcinoma cells [48–50]. HLA-G protein level was elevated in breast cancer cells in response to estradiol administration [51]. Likewise, prolactin exhibited the same promoting effect on trophoblast cells [52]. The low expression of HLA-G in trophoblast cells has been validated to be a contributing factor to recurrent miscarriage, whereas glucocorticoids could potently reverse the phenomenon and enhance HLA-G expression in a concentration-dependent manner, hinting that glucocorticoids might be beneficial to protecting the fetus from immune system attack in patients with recurrent miscarriage [53]. Moreover, mounting studies have shown that epidermal growth factor (EGF), interferons (IFN-α, IFN-β, IFN-γ), interleukins (IL-1β, IL-2, IL-4, IL-10), transforming growth factor (TGF-β) and tumor necrosis factor (TNF-α) could regulate HLA-G expression [52, 54–64]. It is noteworthy that in certain HLA-G-negative cell lines, IFN-γ failed to upregulate HLA-G levels but could further amplify 5-Aza-induced HLA-G expression, hinting an important role of IFN-γ in maintaining HLA-G expression. Notably, IFN-γ failed to upregulate HLA-G levels in some HLA-G-negative cell lines [65, 66].

Studies on HLA-G expression being modulated by aberrant signaling pathways are limited. Chemotherapy upregulated HLA-G by inhibiting DNMT1 and inducing demethylation of TAP1, providing opportunities for anti-HLA-G CAR-NK cells to eliminate cancer cells [22]. More recent research demonstrated that EGFR promoted IL-1β secretion via NLRP3 inflammasome to induce HLA-G expression, resulting in immunosuppression in oral squamous cell carcinoma (OSCC) [11].

### HLA-G expression and clinical significance in cancer

HLA-G is engaged in the evolution of normal tissue cells and their transformation into malignant cells, and its expression and polymorphisms have been linked to precancerous lesions and cancer susceptibility. Long-term and persistent infection with high-risk HPV viruses would result in cervical intraepithelial neoplasia (CIN), approximately 30–40% of which will progress to invasive cervical cancer [67, 68]. Growing studies have suggested that HLA-G 3'UTR polymorphisms are likely to be independent risk factors for HPV infection, as indicated by the intimate association between HLA-G 14bp In/ + 3142G/ + 3142C allele and susceptibility to HPV and the progression of cervical lesions [69]. It has also been shown that amplification of HLA-G + 3142C allele was a contributing factor to sHLA-G upregulation and significantly correlated with susceptibility to HPV [70–73]. In addition, HLA-G*01:01:02/01:01:08 alleles and HLA-G*01:04:01 homozygote were associated with increased risk and decreased risk of infection, respectively [74, 75]. HLA-G*01:01:02 and HLA-G*01:03 alleles were found to be significantly related to persistent HPV16 infection [75]. HLA-G has been implicated in malignant transformation [76], increasing throughout CIN progression [77–79] and peaking in cervical cancer [79–81]. Along similar lines, in hepatocellular carcinoma, the HLA-G 14bp I/D polymorphism was significantly correlated with tumor development in HBV/HCV( +) cases rather than in the HBV/HCV(-) subset [82]. A large-scale GWAS study identified HLA-G as a principal locus related to the risk of CRC [83], with DelG haplotype and InsC haplotype indicating an increased or decreased risk of developing CRC, respectively [84]. In breast cancer, HLA-G + 3142G was a protective factor for cancer susceptibility, while the + 3142C allele acted in the opposite way [85]. 14bp Del and + 3010/ + 3142/ + 3187 variants were found to be of great value in distinguishing breast cancer patients from the general population [86]. Similarly, HLA-G 14bp Del allele and 14bp Del/ + 3142 C variants displayed a higher frequency in patients with gastric cancer [87]. Thus, HLA-G polymorphic variants are closely related to the development or progression of several malignant pathologies.

**Fig. 2:**
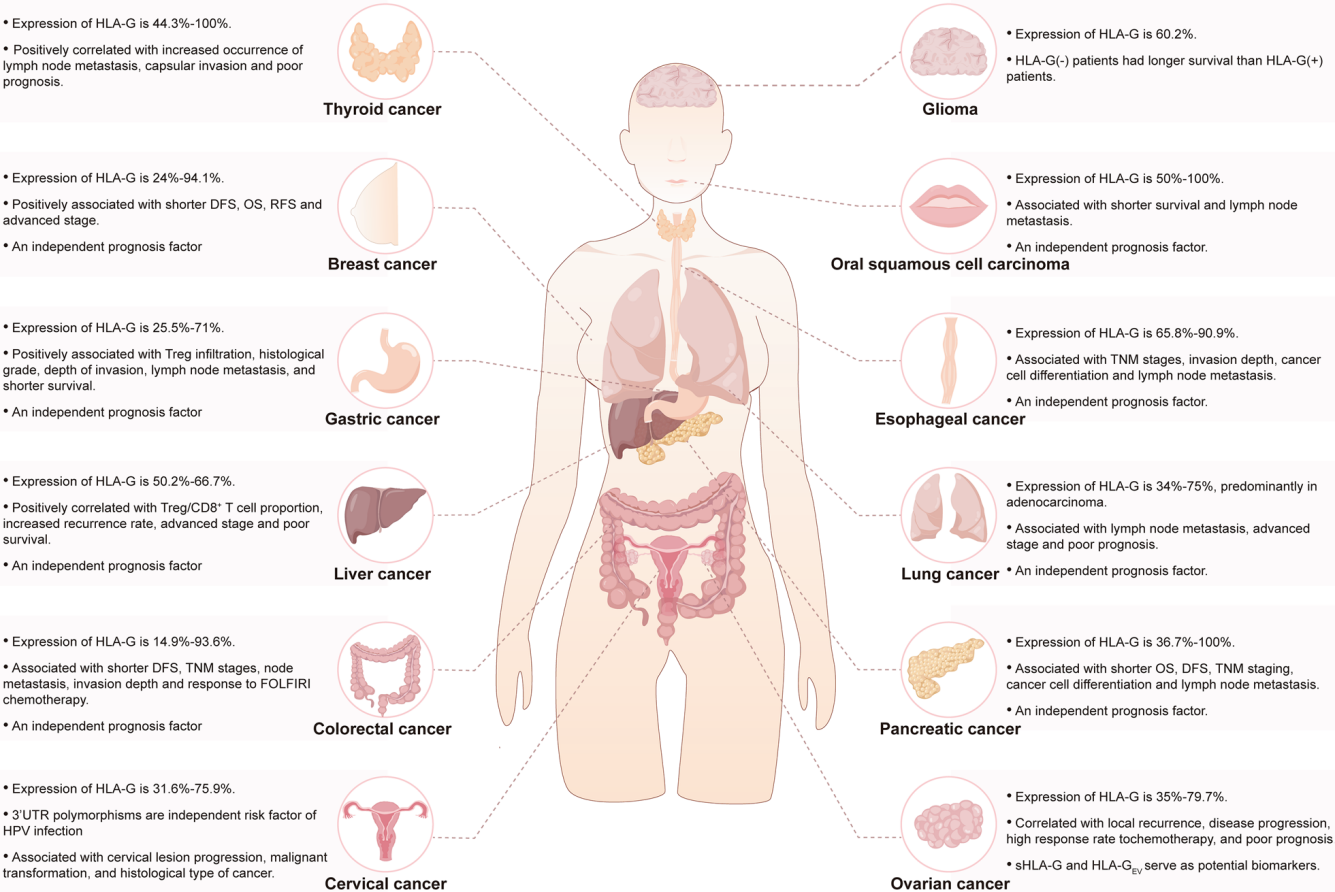
A schematic presentation of expression and clinical significance of HLA-G in cancers. HLA-G is aberrantly enriched in various cancers and associated with clinical characteristics and prognosis

HLA-G was aberrantly enriched in a variety of tumors (Fig. 2), including colorectal cancer [88], gastric cancer [89], ovarian cancer [90–93], thyroid cancer [40], cervical cancer [78, 94], and endometrial cancer [95–97]. In colorectal cancer, HLA-G was observed to aggregate at the invasion front in cancer foci, which was conducive to the formation of an immunosuppressive microenvironment, favoring further invasion and metastasis [10]. It is desirable to explore whether this phenomenon is universal in pan-cancer. HLA-G exhibited a strong co-localization with CA125 in ovarian cancer [98], indicating that HLA-G might be a valuable predictive biomarker for early ovarian cancer. HLA-G expression has been reported to be modulated by the grading and staging of the tumor, and HLA-G protein levels were significantly higher in patients with advanced stages in comparison to early-stage and normal [11, 98–102]. Inconsistently, HLA-G expression did not correlate with disease stage in thyroid cancer [103]. HLA-G is suggestive of tumor progression and recurrence as denoted by the positive correlation between HLA-G high-expression and advanced stage [99, 104–107], distant metastasis [108], lymph node metastasis [102, 105, 109], and higher recurrence rates [90, 101, 110]. In ovarian cancer, compared to the tumor cells in situ, metastatic tumor cells in ascites had higher expression of HLA-G, which was inversely correlated to the frequency of immune cell infiltration and positively related to disease progression, tumor metastasis, and poor prognosis [93, 98]. Also, extensive evidence has confirmed that high HLA-G expression was positively related to shorter survival [43, 64, 88, 90, 102, 111–120] and was an independent risk factor for prognosis [51, 115, 121–126]. Recently, an eight-gene prognostic model constructed on the basis of HLA-G-driven differential genes demonstrated good predictive value for the prognosis of cervical cancer patients (AUC = 0.896) [127]. HLA-G may also function as a biomarker for screening potential benefit populations and evaluating therapeutic efficacy, as exemplified by a substantial reduction of HLA-G expression after chemotherapy administration, which probably arises from the increased sensitivity to chemotherapy of HLA-G^+^ tumor cells [128], and a higher response rate to chemotherapy in ovarian cancer patients with high HLA-G expression compared to the counterparts with low HLA-G expression [129]. Similarly, HLA-G receptor ILT2 was related to worse adjuvant chemotherapy responses and indicative of poor response to anti-PD-1/PD-L1 therapies when combined with the absence of CD8^+^T cells [130]. In colorectal cancer, HLA-G allele could be used to screen patients who might show a good response to first-line FOLFIRI chemotherapy regimens, as the response rate compared favorably in patients carrying + 3010G and + 3187G allele with that seen in patients carrying + 2960-Ins allele [131]. Collectively, the above results indicate that HLA-G has potential clinical implications in guiding the treatment of cancer patients, and may serve as a potential diagnostic and prognostic biomarker.

Intriguingly, sHLA-G in circulation has fueled the tumor cells to spread immunosuppressive signals. Elevated levels of sHLA-G have been observed in gastric cancer, ovarian cancer, cervical cancer, and endometrial cancer, hinting at the potential of sHLA-G in distinguishing malignant from benign and defining tumor stages [82, 91, 132–137]. Besides, serum sHLA-G levels exhibited remarkable diagnostic value for thyroid cancer (AUC = 0.925), which was linked to clinicopathological features including tumor size, lymph node metastasis, and degree of differentiation, and showed significant negative correlation with 1 year and 3 year survival rates [138]. In colorectal cancer, sHLA-G levels in plasma were positively correlated with the number of metastatic sites [139, 140] and inversely related to survival [108], whereas high levels of sHLA-G in saliva were suggestive of later disease stage [141]. High levels of plasma sHLA-G were found in patients with locally advanced breast cancer [142], rather than in the triple-negative breast cancer (TNBC) subgroup [143, 144]. This difference may be attributed to the lack of hormone receptors, as suggested by experimental studies that have shown elevated levels of sHLA-G in hormone-receptor-positive patients compared to their negative counterparts [51]. A recent study revealed that in TNBC patients, sHLA-G plasma levels were significantly increased after neo-adjuvant and adjuvant chemotherapy compared with pre-treatment, and were associated with distant metastases and poorer disease outcomes [144]. In contrast, plasma sHLA-G dramatically declined within 30 days after radical hysterectomy [145]. Hence, whether monitoring the dynamic changes of sHLA-G in plasma could be employed in evaluating efficacy and predicting recurrence in tumor patients after surgery or chemotherapy warrants further investigation. Moreover, increased amount of HLA-G-bearing extracellular vesicles (HLA-G_EV_) predisposed epithelial ovarian cancer patients to disease progression and poor prognosis, being indicative of higher disease risks [146]. Recently, HLA-G protein expression on the membrane of exosomes has also been confirmed in gastric cancer [136]. Compared with tissue biopsies for intra-tumor HLA-G expression, liquid biopsies for analysis of HLA-G_EV_ are easy in operation, cost-effective, and noninvasive. It is reasonable to speculate that the measurement of HLA-G_EV_ in combination with sHLA-G might be a supplement to tumor markers in cancer screening.

In conclusion, owing to the heterogeneity of the tumors and the discrepancies in detection tools and reagents, studies regarding HLA-G expression in cancers have shown mixed results (Table 2). Therefore, it is imperative to conduct additional pre-clinical and clinical research to pinpoint the role of HLA-G in precancerous and malignant lesions. This will help advance the potential of HLA-G as a predictive biomarker for early cancers from bench to bedside.

**Table 2:** Expression and clinical features of HLA-G in different tumors

Tumor	HLA-G expression, Method	Clinical implication	References
Breast cancer	94.1%, IHC	Positively associated with shorter OS and RFS	[244]
Breast cancer	70.7%, IHC	Positively associated with advanced disease stage	[107]
Breast cancer	66%, IHC	An independent prognosis factor	[51]
Breast cancer	62.2%, IHC	Positively associated with shorter survival	[111]
Breast cancer	41%, IHC	Positively associated with shorter DFS	[114]
Breast cancer	24%, IHC	Not associated with clinical outcome	[245]
Breast cancer	Not mentioned	14-bp Ins/Del and + 3142 C/G are associated with breast cancer susceptibility	[85]
Breast cancer	Not mentioned	HLA-G 14bp Del and the + 3010/ + 3142/ + 3187 variants are more prevalent in breast cancer patients than in controls	[86]
Breast cancer	Median sHLA-G plasma level 41.3 ng/ml, ELISA	A higher level of sHLA-G before NACT is related to disease progression	[142]
Triple negative breast cancer (TNBC)	Median sHLA-G plasma level 8.6 ng/ml, ELISA	High post-chemotherapy sHLA-G levels were associated with the development of distant metastases and poorer disease outcomes. The combination of high sHLA-G levels post-chemotherapy and ILT-2 rs10416697C allele carrier status is a better independent indicator for disease outcome in TNBC than the lymph nodal status pre-chemotherapy	[144]
Cervical cancer	75.9%, IHC	An early marker for disease progression	[78]
Cervical cancer	45%, IHC	Association with the size of the main lesion, parametrial invasion, and lymph node metastasis	[137]
Cervical cancer	35.7% in CIN, 62.8% in SCC, IHC	Positively associated with disease progression	[80]
Cervical cancer	31.6%, IHC	Low expression of HLA-G5 is detected in all HPV-related cases	[94]
Cervical cancer	Not mentioned	14bp In/ + 3142G/ + 3142C allele is associated with susceptibility to HPV and the progression of cervical lesions	[69]
Cervical cancer	Not mentioned	+ 3142 C/C genotype and C allele are associated with increased risk	[71]
Cervical cancer	Not mentioned	14 bp del allele promotes high-risk HPV infection, del/C haplotype is associated with invasive cervical cancer development	[72]
Cervical cancer	Not mentioned	HLA-G*01:01:02 and HLA-G*01:03 alleles are related to persistent HPV16 infection	[75]
Cervical cancer	Not mentioned	14 bp In and + 3142G are increased in the HPV18-infected group compared with the control. HLA-G expression increases from CIN1 to CIN2/3 lesions and is highest in patients with adenocarcinoma	[77]
Cervical cancer	12.5% in CIN 1, 35.9% in CIN 2/3, 78% in cervical cancer, IHC	HLA-G expression increases from CIN 1 to CIN 2/3 and is highest in patients with cervical cancer. HLA-G expression is higher in CIN and cancer patients with HPV 16/18 than in CIN patients without HPV	[79]
Cervical cancer	Median sHLA-G plasma level 115.8 U/ml, ELISA	sHLA-G level could be used as a diagnostic marker	[132]
Cervical cancer	Median sHLA-G plasma level 50.86 U/ml, ELISA	sHLA-G level could be used as a diagnostic marker and decreases within 30 days after radical hysterectomy	[145]
Colorectal cancer	70.7%, IHC	HLA-G expression above 55% was associated with a worse prognosis	[120]
Colorectal cancer	70.6%, IHC	Associated with the OS, an independent factor for OS	[139]
Colorectal cancer	64.6%, IHC	Associated with the depth of invasion, histological grade, host immune response, lymph nodal metastasis, and clinical stages of the disease	[124]
Colorectal cancer	59.1%, IHC	Associated with tumor node metastasis staging	[105]
Colorectal cancer	Median sHLA-G plasma level 124.3 U/ml, ELISA	sHLA-G level is higher in colorectal cancer and may be a diagnostic marker	[246]
Colorectal cancer	14.9% in EpCAM^+^ cancer cells, Flow cytometry	Associated with lymph node metastasis and shorter OS	[102]
Colorectal cancer	Not mentioned	HLA-G DelG haplotype is associated with increased cancer risk, while InsC haplotype is associated with decreased risk. InsC haplotype is independently associated with longer EFS	[84]
Colorectal cancer	Median sHLA-G plasma level 59.17 U/ml, ELISA	sHLA-G level is correlated with HLA-G 3’UTR polymorphisms/haplotypes. Patients carrying + 3010G and + 3187G alleles have a higher chance of complete response to first-line FOLFIRI treatment	[131]
Colorectal cancer	Median sHLA-G plasma level 36.8 U/ml, ELISA	sHLA-G above median levels (≥ 36.8 U/ml, sHLA-G_high_) had a shorter survival time than those with sHLA-Gl_ow_ (< 36.8 U/ml,), an independent prognostic factor	[108]
Colorectal cancer	Median sHLA-G salivary level 18.84 U/ml, ELISA	sHLA-G salivary level is associated with advanced stages and sHLA-G serous level	[141]
Colon cancer	22.1%, IHC	Positively associated with shorter OS and DFS	[247]
Esophageal cancer	90%, IHC	Correlated with histologic grade, depth of invasion, nodal status, host immune response, clinical stage of disease, and worse prognosis, an independent prognostic factor	[248]
Esophageal cancer	70%, IHC; median sHLA-G plasma level 15.04 U/ml, ELISA	Associated with cancer cell differentiation, lymph node metastasis, and poor prognosis	[109]
Esophageal cancer	65.8%, IHC; median sHLA-G plasma level 152.4 U/ml, ELISA	Associated with advanced disease stage and poor survival, an independent prognostic factor	[126]
Gastric cancer	71%, IHC	Associated with the tumor location, histological grade, depth of invasion, lymph nodal metastasis, clinical stages of the disease, host immune response, and shorter OS, an independent prognostic factor	[249]
Gastric cancer	49.7%, IHC	Associated with the number of tumor-infiltrating Tregs, tumor invasion depth, invaded adjacent organs, clinical stages, and poorer prognosis (OS, DFS, and cancer-specific survival), an independent prognostic factor	[250]
Gastric cancer	30.8%, IHC	Positively associated with the number of tumor-infiltrating Tregs and negatively associated with the number of CD8 + T lymphocytes, an independent prognostic factor	[251]
Gastric cancer	25.5%, IHC	Positively associated with shorter OS	[117]
Gastric cancer	Not mentioned	HLA-G 14bp Del allele and the 14bp Del/ + 3142 C variants are increased in patients with gastric cancer. The survival rate in patients bearing the 14bp DelL/Del genotype is lower than in patients with either Ins/Del or Ins/Ins genotypes	[87]
Glioma	60.2%, IHC	The absence of HLA-G expression is associated with a better long-term survival rate	[21]
Liver cancer	43% low expression, 57% high expression, IHC	Positively associated with Tregs/CD8 + ratio and shorter OS	[101]
Liver cancer	50.2%, IHC; median sHLA-G plasma level 92.49 U/ml, ELISA	Associated with advanced disease stage	[104]
Liver cancer	median sHLA-G plasma level 178.8 U/ml, ELISA	Serum sHLA-G levels could be used as a diagnostic marker	[252]
Liver cancer	Not mentioned	HLA-G 14bp I/D polymorphism is significantly correlated with tumor development in HBV/HCV( +) cases rather than in the HBV/HCV(-) subset	[82]
Lung cancer	75%, IHC	Associated with lymph nodal metastasis, clinical stages of the disease, host immune response, and shorter OS, an independent prognostic factor	[123]
Lung cancer	41.6%, IHC; median sHLA-G plasma level 34 U/ml, ELISA	HLA-G expression and plasma sHLA-G level are associated with disease stages and shorter survival	[99]
Lung cancer	34%, IHC; median sHLA-G plasma level 23.6 U/ml, ELISA	sHLA-G expression is higher in adenocarcinoma lesions than in squamous cell carcinoma and adenosquamous carcinoma lesions	[253]
Lung cancer	51.85%, IHC	Co-expression of ILT4/HLA-G is associated with regional lymph node involvement, advanced stages, and shorter OS	[121]
Lung cancer	Median sHLA-G plasma level 53.3 U/ml, ELISA	Patients with the sHLA-G above median level (≥ 50 U/ml) have a significantly shorter survival time	[254]
Oral squamous cell carcinoma	50%, IHC	Positively associated with shorter OS	[255]
Oral squamous cell carcinoma	18.2% low expression, 81.8% high expression, IHC	Associated with clinical tumor stage and shorter OS	[118]
Ovarian cancer	72.4%, IHC	Associated with disease recurrence	[90]
Ovarian cancer	61%, IHC	sHLA-G level is higher in malignant as compared with benign ascites and could be used as a diagnostic marker	[91]
Ovarian cancer	79.7%, IHC	HLA-G5/-G6 expression was detected in 75.7% ovarian serous cancer, 63.6% mucinous cystadenocarcinoma and 100% endometrioid adenocarcinoma, 85.7% clear cell carcinoma, 100% sex cord-stromal tumor and 77.8% germ cell tumors	[92]
Ovarian cancer	35%, IHC	Associated with high-grade histology	[98]
Ovarian cancer	55% low expression, 20% median expression, 25% high expression	Associated with CA125 elevation and shorter OS	[113]
Pancreatic cancer	36.1% low expression, 63.9% high expression, IHC; median sHLA-G plasma levels 70.56 U/ml, ELISA	Associated with advanced stage, extra-pancreatic infiltration, lymph node involvement, and poor differentiation, an independent predictor for OS	[115]
Pancreatic cancer	39.2%, IHC	Positively associated with T stage and shorter OS	[256]
Pancreatic cancer	66%, IHC	Associated with advanced stages and grades	[106]
Pancreatic cancer	36.7%, IHC	Positively associated with shorter OS and DFS	[64]
Thyroid cancer	3% low expression, 17% median expression, 80% high expression in papillary thyroid carcinoma	In papillary thyroid carcinoma, the percentage of tumor cells exhibiting strong HLA-G staining is higher in patients with tumor size > 1.0 cm when compared to lesions < 1 cm	[103]
Thyroid cancer	Median sHLA-G plasma level 24.84 ng/mL, ELISA	sHLA-G level is associated with tumor size, differentiation degree, capsule invasion, lymph node metastasis	[138]
Papillary thyroid carcinoma	44.3%, IHC	Associated with lymph node metastasis and capsular invasion	[257]
Papillary thyroid carcinoma	Median sHLA-G plasma level 42.9 ng/mL, ELISA	sHLA-G level was higher in papillary thyroid carcinoma than that in healthy controls	[258]

*IHC* immunohistochemistry, *OS* overall survival, *DFS* disease-free survival, *RFS* relapse-free survival., *EFS* event free survival, *NACT* neoadjuvant chemotherapy, *ELISA* enzyme linked immunosorbent assay, *CIN* cervical intraepithelial neoplasia, *HPV* human papilloma virus, *HBV* hepatitis B virus

### HLA-G interplay with immune receptors

Several receptors for HLA-G have been identified, including ILT2, ILT4, and KIR2DL4. ILT-2 and ILT-4 are the members of the leukocyte immunoglobulin-like receptor (LILR) family. Each consists of four extracellular Ig-like domains, with D1D2 responsible for HLA-G binding and D3D4 acting as a scaffold [147]. ILT2 and ILT4 both have a cytoplasmic tail that contains immunoreceptor tyrosine-based inhibitory motifs (ITIM) that interact with tyrosine phosphatases (SHP1/SHP2) and initiate inhibitory signaling [148]. It has been revealed that the ILTs have a broad specificity for MHC class I molecules, but they exhibit the highest affinity for HLA-G [149]. Moreover, the binding affinity of HLA-G to ILTs was improved by the dimerization of HLA-G due to the increased exposure of binding site [147, 150]. ILT2 is expressed on various immune cells, including T cells, B cells, NK cells, myeloid-derived suppressive cells (MDSCs), dendritic cells (DCs), and monocytes/macrophages, whereas ILT4 is exclusively presented on DCs, monocytes/macrophages, neutrophils and MDSCs [151–155]. KIR2DL4 belongs to the killer cell immunoglobulin (Ig) like receptor (KIR) family, with a charged arginine residue near the top of its transmembrane region that can associate with the immunoreceptor tyrosine-based activating motif (ITAM). It also has a single intracellular ITIM domain and exhibits weak inhibitory potential [156]. Due to its unique structure, KIR2DL4 functions as both an activating and inhibitory receptor. KIR2DL4 lacks a D1 domain, and its interaction with HLA-G is mediated by the D0 or D2 domain [157]. In contrast to ILT2/4, which is expressed in a wide range of cells, KIR2DL4 is predominantly found on NK cells [156]. A recent study demonstrated that KIR2DL4 synergized with FcRγ to augment NK cell activation and degranulation, whereas the interaction of HLA-G with KIR2DL4 attenuated NK cell cytotoxicity in HER2-positive breast cancer [158]. Other receptors, such as CD160, are expressed by endothelial cells, and the interaction between HLA-G and CD160 can lead to apoptosis and thus inhibit angiogenesis [159]. In addition, it has been reported that HLA-G binding to CD8 modulates the activation and apoptosis of cytotoxic T cells and NK cells [160–162].

### HLA-G functions on immune cells

**Fig. 3:**
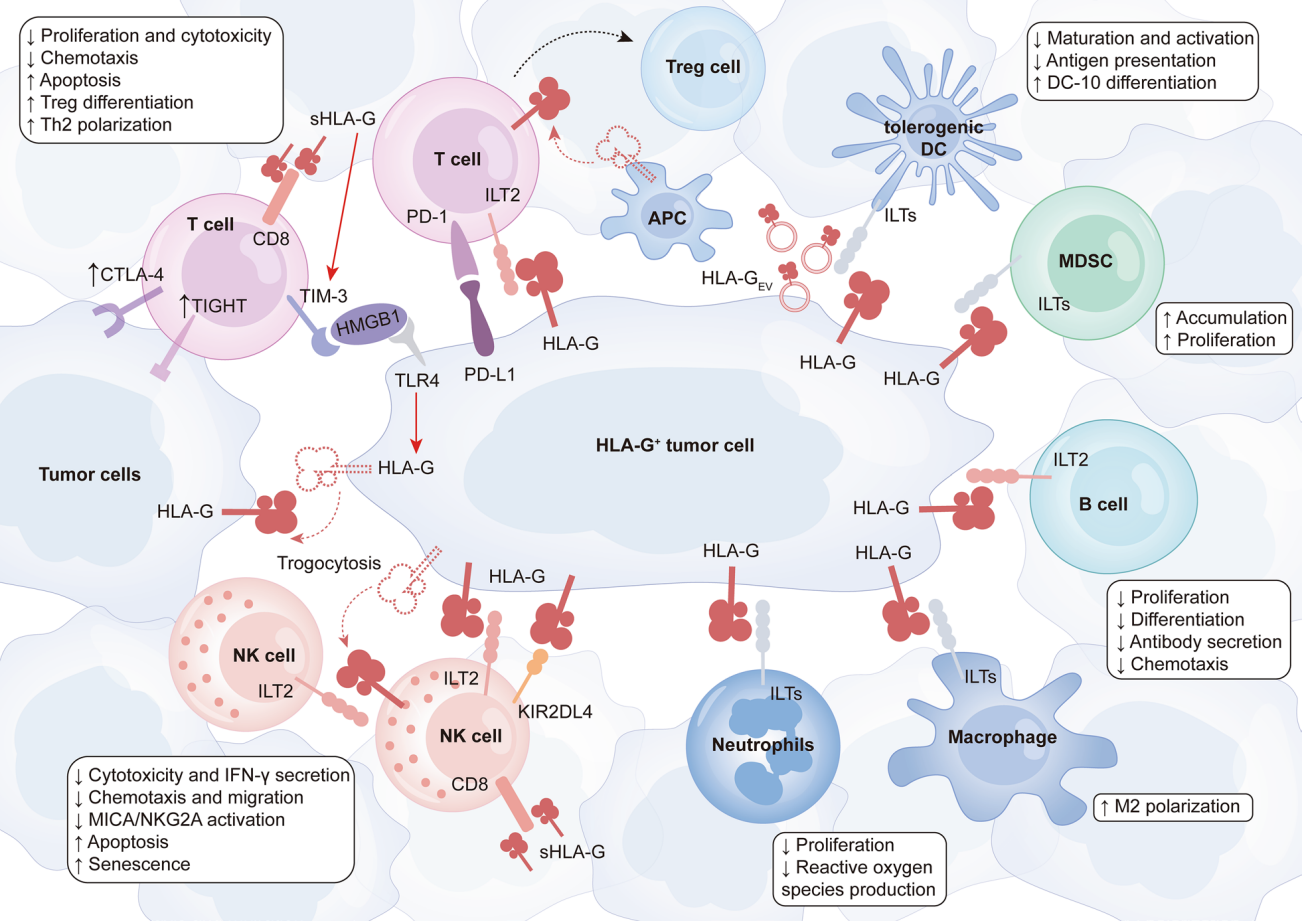
Roles of HLA-G in tumor immune microenvironment. HLA-G can inhibit the cytotoxicity and chemotaxis of T cells and foster the differentiation of the CD4 + T cells into Treg cells through direct binding, while sHLA-G can drive Th2 cells polarization and potentiate TIM-3 expression to indirectly exert suppressive effects on T cells. Similarly, HLA-G dampens NK cells cytotoxicity, chemotaxis, and migration, while inducing apoptosis and senescence. Moreover, the interaction between HLA-G and ILTs restrains the proliferation of neutrophils, impairs the proliferation, differentiation, chemotaxis, and antibody secretion of B cells, and triggers a shift in macrophage polarization toward M2. MDSC proliferation and accumulation are facilitated by HLA-G. Additionally, HLA-G impairs the activation and antigen-presenting function of DCs and promotes tolerogenic DCs induction. To note, tumor cells can transfer HLA-G to other tumor cells and effector immune cells, leading to the swift dissemination of immunosuppression. *NK cells* natural killer NK cells, *MDSC* myeloid-derived suppressor cell, *DCs* dendritic cells

Broad evidence has suggested that HLA-G could modulate innate and adaptive immunity through its interaction with ILT2/4 and KIR2DL4 (Fig. 3). HLA-G restrained CD4^+^ and CD8^+^ T cells proliferation [163, 164], and concomitantly induced apoptosis through Fas/FasL signaling pathway via CD8 ligation [160, 162]. Furthermore, HLA-G fostered the differentiation of the CD4 + T cells into Treg cells [165, 166], while HLA-G1-induced CD4^+^ T cells would enter a long-term unresponsive state to the specific immunity, diffusion of which might cause a wider range of immune tolerance [167]. HLA-G directly impaired the cytotoxicity of effector T cells [168], mitigating the anti-tumor activity of γδΤ cells [169] and facilitating the immune evasion of target cells. Additionally, the chemotaxis of T cells was affected by sHLA-G via different mechanisms. sHLA-G reduced CXCR3 expression on CD8^+^ T cells and γδΤ cells to dampen the response to CXCL10 and CXCL11-mediated chemotaxis. The chemotaxis of CD4^+^ T cells induced by CCL2, CCL8, CXCL10, and CXCL11 was severely impaired due to the downregulation of CCR2, CXCR3, and CXCR5, whereas the recruitment of follicular helper T cells (T_FH_) to CXCL13-expressing regions was hindered on account of decreased CXCR5 expression [170]. To note, sHLA-G derived from macrophages or activated monocytes could drive the polarization of T helper 2 (Th2) cells and promote the secretion of IL-10, IL-4, and IL-3, which in turn further upregulated HLA-G expression. Such positive feedback disrupted the natural equilibrium between Th1 and Th2, leading to a severe immunosuppressive state [171].

NK cells are blessed with powerful non-specific killing capacity and are integral to the innate immune system. In analogy to T cells, HLA-G is implicated in the suppression of cytotoxic effects of NK cells [172–174] and is capable of reducing the secretion of IFN-γ substantially [175]. HLA-G, especially HLA-G1 and HLA-G3, could boost HLA-E expression [176, 177] and potently suppress NK cells function via HLA-E/NKG2A interaction [178]. Simultaneously, the inhibitory impact of HLA-G was amplified synergistically due to the mitigation of NK cell activation signal MICA/NKG2D [179]. It has been validated that HLA-G induced over-expression of ILT2, ILT3, ILT4, and KIR2DL4 on NK cells independent of antigen stimulation, which might upregulate the threshold of NK cells activation, favoring the immune evasion of target cells [180]. Moreover, the trans-endothelial migration of NK cells was hindered once HLA-G bound with ILT2 [181]. sHLA-G also decreased CXCR3, CCR2, and CX3CR1 expression on peripheral blood NK cells, impeding their chemotactic responses to CXCL10, CXCL11, CCL2, and CX3CL1 and restraining their recruitment to the lesion [182]. On the other hand, sHLA-G fostered FasL expression and secretion, which triggered apoptosis of CD8^+^ NK cells [162].

In addition, HLA-G suppressed B cells proliferation, differentiation, and antibody secretion after binding to ILT2, and decreased CXCR4 and CXCR5 expression on B cells in the germinal center to restrict the chemotaxis [183, 184]. Additionally, exposure to sHLA-G5 triggered macrophage polarization towards M2 as evidenced by an increase in CD163 expression and a decrease in CD86 expression [185]. HLA-G modulated DCs differentiation via IL-6/STAT3 signaling pathway [186], fostering the induction of tolerogenic DCs while impairing their activation and antigen-presentation functions [187, 188]. In particular, the tolerogenic subset DC-10 could overexpress HLA-G to induce the generation of Treg cells dependent on IL-10 and inhibit cytokine secretion of activated invariant natural killer T (iNKT) cells, contributing to immune response suppression. In this setting, it is plausible that HLA-G could amplify the immune escape effect with the help of the positive feedback loop formed by DCs [151, 189]. Moreover, the expansion and accumulation of MDSCs were also remarkably promoted in response to HLA-G [153, 190–192]. Collectively, HLA-G could impede the functions of effector cells while enhance the activities of suppressive and regulatory cells, supporting the remodeling of the immunosuppressive microenvironment. However, there is an ongoing need for studies regarding the clinical significance of the crosstalk between HLA-G and immune cells, which will aid in the development of effective treatment schemes.

### HLA-G interaction with immune checkpoint molecules

Immune checkpoints are a group of immunosuppressive molecules expressed on the surface of immune cells, which are dedicated to regulating immune response and essential for the maintenance of autoimmune tolerance and homeostasis. Nevertheless, immune checkpoint molecules are exploited by tumor cells to impede the tumor-killing effects of immune cells with the goal of evading immune attack. As discussed above, growing evidence has demonstrated that HLA-G could potently blunt the cytotoxicity of immune cells including T cells and NK cells, thus its potential as a novel immune checkpoint is gaining increased attention. However, the discrepancies and connections between HLA-G and other known immune checkpoint molecules remain obscure.

1. PD-L1

Programmed death-ligand 1 (PD-L1), is a 40 kDa transmembrane protein encoded by *CD274* gene, representing a crucial suppressor of anti-tumor immunity [193]. HLA-G and PD-L1 exhibited consistent high-expression in pancreatic cancer [64], breast cancer [194], papillary thyroid cancer [195], kidney cancer [12, 196, 197], colorectal cancer [102], oral osteosarcomas [198], and intraoral mucoepidermoid carcinoma [199]. In ovarian and gastric cancer, differentiating agents rendered tumor cells more sensitive to chemotherapy drugs due to the loss of stemness and proliferation capacity. However, expression of HLA-G and PD-L1 was upregulated concomitantly, reshaping the suppressive tumor immune microenvironment, which might be a contributor to the poor efficacy of such drugs [200]. Trastuzumab-induced TGF-β and IFN-γ also facilitated HLA-G and PD-L1 expression on HER2-positive breast cancer cells, contributing to trastuzumab resistance [158]. In non-small cell lung cancer (NSCLC), HLA-G and PD-L1 expression were boosted in a dose-dependent manner by several chemotherapeutic agents, among which pemetrexed promoted the glycosylation of HLA-G and PD-L1 consistently as a belt-and-braces approach to the tumor cell immune escape [201]. Despite the sizable evidence implicating a pronounced overlap in the expression patterns of HLA-G and PD-L1 across different tumors, there is heterogeneity in the areas of expression and levels of expression within the same tumor [196].

Intriguingly, HLA-G and PD-L1 have been reported to share other similarities. Firstly, both could induce apoptosis, namely, sHLA-G binding to CD8 triggered apoptosis in T cells and NK cells through Fas/sFasL pathway [162], whilst PD-L1 expressed on lymphatic endothelial cells (LECs) could elicit apoptosis in tumor-specific CD8^+^ central memory T cells [202]. These results indicated that both HLA-G and PD-L1 could not only directly reduce the population of effector immune cells, but also restrain the lymphocytes functions by engaging with corresponding receptors, working along both lines to shield tumor cells from immune killing. Secondly, both PD-L1 and HLA-G exist in the form of exosomes to impede immune cell functions. Exosomes are nano-vesicles released by normal or neoplastic cells, containing proteins, metabolites, and other bioactive molecules, participating in physiological processes such as tumor angiogenesis, immune evasion, and long-distance intercellular communication [203]. Exosomal PD-L1 inhibited the proliferation of CD4^+^ and CD8^+^ T cells, decreased the abundance of T cells in spleen and lymph nodes, and impaired the NK cells cytotoxicity characterized by reduced secretion of IL-2, IFN-γ, and Granzyme B [204]. Exosomal PD-L1 is more effective than soluble PD-L1 in inducing systemic immunosuppression due to its rich circulating routes and robust inhibitory effect, contributing to the poor response rate to anti-PD-L1 therapy and development of drug resistance in most patients [203]. Indeed, PD-L1-targeted therapy in combination with the inhibition of exosome secretion using genetic means or small molecule drugs has shown promising therapeutic efficacy [205–207]. In 2003, Be´atrice Riteau et al. reported the existence of secretory exosomal form of HLA-G for the first time [208], whereas the exosomal HLA-G has not been identified in ascitic and pleural exudates from cancer patients until 2013 [44]. To date, the origin of exosomal HLA-G remains inconclusive, but a previous study suggested it might be derived from stem cell-like cancer cells in renal cancer [209]. Differentiation and maturation of peripheral blood mononuclear cell (PBMC)-derived DC cells were restrained by exosomal HLA-G, as well as the T cells activation mediated by DC cells [209]. However, whether exosomal HLA-G has the exact same immunosuppressive functions as membrane-bound HLA-G remains to be appraised. In addition, higher level of exosomal HLA-G was coupled to the presence of stem cell-like circulating tumor cells and the resulting disease progression and poor prognosis, hinting that modalities targeting exosome inhibition might serve as amplifiers of existing neoadjuvant chemotherapy to benefit breast cancer patients [142]. Finally, both HLA-G and PD-L1 proteins could be transferred intercellularly. Trogocytosis was defined as the transfer of membranal molecules from one cell to another, typically through immune synapses [210]. CD8^+^ T cells acquired functionally active PD-L1 from neighboring mature DC cells or tumor cells via trogocytosis, rendering them into the fight with surrounding PD1^+^ T cells and resulting in the diffusion of local immunosuppression [211]. The pattern of HLA-G trogocytosis was more diversified and characterized by rapid, transient, and intercellular contact-dependent. Tumor cells were able to deliver HLA-G to effector immune cells. NK cells that received HLA-G from melanoma cells lost their proliferation and tumor-killing capacity and suppressed other NK cells cytotoxicity locally and transiently [212], while the occurrence of CD3^+^HLA-G^acq+^T cells was significantly correlated with poor prognosis in multiple myeloma [213]. Some activated and resting T cells obtained HLA-G from antigen presenting cells (APCs) by trogocytosis and then transformed into Treg cells, acquiring immunosuppressive functions comparable to those of natural Treg cells [214]. Based on the above results, trogocytosis has been regarded as an emergency response to immune attack for HLA-G-expressing cells and tissues. The extensive immunosuppression was orchestrated due to the rapid spread of HLA-G, which was originally restricted in expression, to more kinds of cells and larger areas, enhancing the capacity of the whole tumor to counteract the immune system. In general, HLA-G and PD-L1 shared some similarities, including undergoing multifaceted and multilayered regulation, being in a process of dynamic changes, having similar intercellular transfer pathways, and exerting potent immunosuppressive functions. Hence, previous immunotherapy paradigms targeting PD-L1 may offer valuable insights for developing treatment options based on HLA-G, however, the characteristics of HLA-G still need to be taken into account.

Notably, a regulatory relationship between PD-L1 and HLA-G was also discovered. Activated CD8^+^T cells pre-treated with sHLA-G exhibited increased level of ILT-2 protein, accompanied by a significant increase in the expression of CTLA-4, PD-1, and TIM3, suggesting a mutual regulation between HLA-G and other immune checkpoints which might be conducive to the immunosuppressive tumor microenvironment (TME) [215].

- 2. Other immune checkpoint molecules

Cytotoxic T-lymphocyte-associated protein 4 (CTLA-4) is one of the first immune checkpoint molecules studied, acting as a transmembrane receptor on T cells that impairs immune response by binding to the corresponding ligands. In BRAF^V600E+^ papillary thyroid cancer, HLA-G, CTLA-4, and PD-L1 expression were consistently high and significantly inversely correlated with thyroid differentiation score [195], coinciding with the data in renal cell carcinoma [216].

T cell Ig and ITIM domain (TIGHT), another novel immune checkpoint that has gained growing attention, interact with CD155/CD122 ligands to participate in the complex immune regulatory network [217]. Limited bioinformatics data suggested that, contrary to consensus, increased expression of HLA-G and TIGHT was linked to longer disease-free and overall survival in TNBC patients [218]. However, further validation is required to confirm this finding.

There is insufficient data to clarify the relationship between HLA-G and TIM-3, except for one report suggesting that administration of sHLA-G or extracellular vesicles carrying HLA-G could potentiate TIM-3 expression [215]. Nevertheless, the upregulation of HLA-G and HMGB1, one of the identified TIM-3 ligands, was induced by IL-1β, and HLA-G could further increase HLA-G protein level in dependence on TLR4 in glioma [219]. Although the binding site of TIM-3 to HMGB1 and its potential impacts are poorly investigated, it is reasonable to conceive that drugs targeting TIM-3 could modulate HLA-G expression via TLR4-HMGB1 axis.

Collectively, there remains scarce information to date about the relationship between HLA-G and immune checkpoints. In-depth demonstration is fundamental for better targets selection in combination with HLA-G blockade strategies.

### Therapeutics targeting HLA-G in cancer

**Fig. 4:**
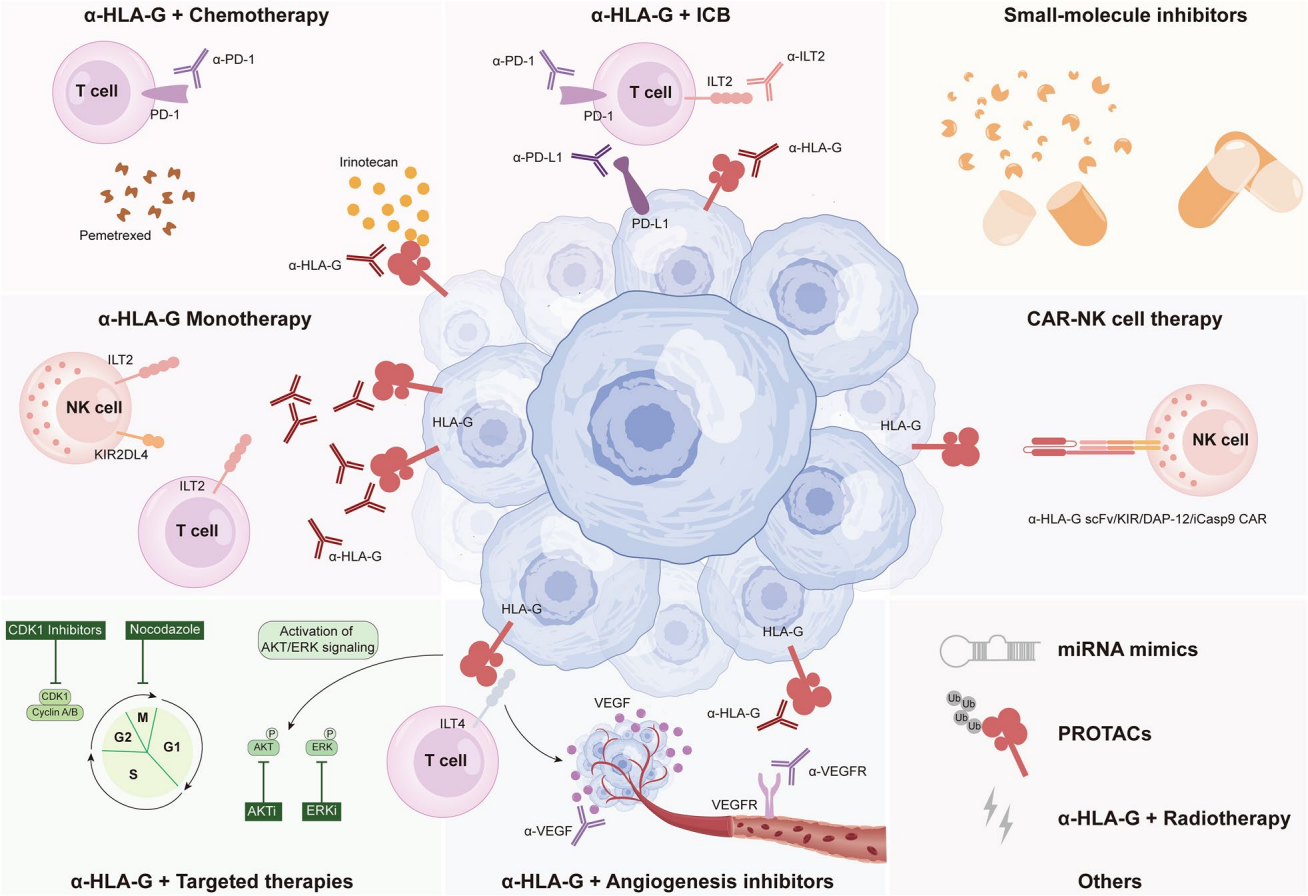
Harnessing HLA-G in cancer immunotherapy. Numerous HLA-G-based therapeutics currently in preclinical or clinical stages could potentially be leveraged to target human cancers. Monoclonal antibodies and CAR-NK cells against HLA-G can restrain tumor growth. Anti-HLA-G antibody also serve as a favorable partner for chemotherapy, ICB, and targeted therapies, including CDKi, ERKi, AKTi, and angiogenesis inhibitors. Moreover, small-molecule inhibitors, miRNA mimics, and PROTACs may provide new opportunities for future applications of HLA-G. *CAR-NK cells* chimeric antigen receptor NK cells, *ICB* immune checkpoint blockade, *CDKi* CDK inhibitors, *ERKi* ERK inhibitors, *AKTi* AKT inhibitors, *PROTACs* proteolysis-targeting chimeras

Immunotherapy represented by anti-PD-1/PD-L1 has revolutionized the paradigm of cancer therapy, however, only about 25% of patients with solid tumors benefit effectively and persistently from anti-PD-1/PD-L1 [7]. Elevated level of sHLA-G in plasma and increased expression of ILT-2 have been implicated as possible mechanisms underlying resistance to anti-PD-1 therapies [220, 221]. In this setting, HLA-G-based therapeutics have emerged, aiming to circumvent the issues. HLA-G highly expressed in renal cancer cells specifically impaired the anti-tumor function of CD8^+^ILT2^+^PD1-T cells, rather than CD8^+^ILT2^−^PBMC or CD8^+^PD-1^+^T cells [222], hinting that blockade of HLA-G might be applicable to the patients who failed to respond to anti-PD-1/PD-L1 therapy, as a complement to existing immunotherapy regimens. The absence of PD-L1 and CTLA-4 and the enrichment of HLA-G in adenoid cystic carcinomas of salivary glands suggested that existing agents targeting PD-L1 or CTLA-4 might be ineffective due to the lack of targets [223]. Instead, targeting HLA-G appears to be a promising therapeutic approach (Fig. 4).

Owing to the heterogeneity and diversity of the cancer, a multitude of clinical trials have focused on combination treatments in order to develop preferable therapeutic regimens with improved efficacy. However, the existing combination strategies, including anti-CTLA-4, chemotherapy, and targeted therapy, have yielded unsatisfactory synergistic effects and severe side effects [7]. Therefore, further studies on the feasibility and clinical application potential of combining anti-PD-1/PD-L1 with HLA-G blockade would be valuable. According to a recent study, aberrantly activated cytokine signaling boosted HLA-G and PD-L1 expression to impair NK cells cytotoxicity, leading to trastuzumab resistance, whereby the immense therapeutic potential of HLA-G-blocking antibody combined with anti-PD-1/PD-L1 antibody in trastuzumab-resistant breast cancer patients was indicated [158].

In addition to the preclinical researches above, clinical trials in small groups are also in progress, summarized in Table 3. A prospective observational cohort study (NCT04300088) explored the impact of HLA-G expression level on the efficacy of tumor immunotherapies (anti-PD-1/PD-L1 therapy and/or anti-CTLA-4 therapy), providing a strong rationale for demonstrating the relationship between HLA-G and tumor resistance to immunotherapy. In another clinical trial for advanced solid tumors (NCT04991740), the effectiveness of a novel bispecific antibody JNJ-78306358 that binds to CD3 and HLA-G has been evaluated, as well as its safety, immunogenicity, and pharmacokinetic properties. BND-22, a monoclonal antibody targeting ILT-2, has been proven to significantly improve the efficacy of anti-PD-1 monotherapy in vitro, and the related phase I/II clinical trials are currently recruiting patients (NCT04717375) [221]. To note, an open-label, dose-escalation/expansion, and multi-center clinical trial (NCT04485013) is enrolling patients with advanced refractory or resistant solid tumors, including head and neck squamous cell carcinoma, colorectal cancer, non-small cell lung cancer, and triple-negative breast cancer, to be given single-agent TTX-080 (anti-HLA-G antibody), or in combination with the PD-1 inhibitor pembrolizumab or the EGFR inhibitor cetuximab. The study aims to determine the safety and tolerability of TTX-080 and illustrate the feasibility and preliminary efficacy of combining TTX-080 with pembrolizumab or cetuximab. This will provide further insight into the future clinical applications of HLA-G inhibition for the benefit of more cancer patients.

**Table 3:** Summary of the clinical trials related to HLA-G

Clinical trial ID	Title	Study phase	Study type	Study design	Participants	Brief summary
NCT04300088	A Prospective Study of the Relevance of the HLA-G Immune Checkpoint in Cancer Immunotherapy (GEIA)	–	Observational	Cohort, prospective	Patients with advanced solid cancer	To explore the impact of HLA-G tumor expression on the efficacy of cancer immunotherapy (anti-PD-1/PD-L1 with or without anti-CTLA4)
NCT04991740	A Study of JNJ-78306358 in Participants With Advanced Stage Solid Tumors	Phase 1	Interventional	Open label, non-randomized, sequential assignment	Patients with RCC, ovarian cancer, CRC, and other types of tumors	To determine the safety, pharmacokinetics, biomarkers, immunogenicity, and efficacy of JNJ-78306358, a bispecific antibody binding to CD3 on T cells and HLA-G on cancer cells
NCT04717375	Study of SAR444881 Administered Alone and in Combination With Other Therapeutics in Participants With Advanced Solid Tumors	Phase 1/2	Interventional	Open label, non-randomized, sequential assignment	Advanced cancer patients with unresectable or metastatic disease who are refractory to or are not candidates for standard approved therapy	To determine the safety, tolerability, and recommended Phase 2 dose of SAR444881 monotherapy and in combination with either pembrolizumab, cetuximab and/or carboplatin and pemetrexed
NCT04485013	TTX-080 HLA-G Antagonist in Subjects With Advanced Cancers	Phase 1a/1b	Interventional	Open label, non-randomized, parallel assignment	Patients with advanced refractory/resistant solid malignancies, including HNSCC, NSCLC, CRC, TNBC, RCC, and acral melanoma	To determine the safety, tolerability, and recommended Phase 2 dose of TTX-080 monotherapy (HLA-G inhibitor) and in combination with either pembrolizumab or cetuximab

*RCC* renal cell carcinoma, *CRC* colorectal cancer, *HNSCC* head and neck squamous cell carcinoma, *NSCLC* non-small cell lung cancer, *TNBC* triple negative breast cancer

Chemotherapeutic agents retard tumor growth chiefly by arresting cell cycle, inhibiting DNA replication, and disturbing cell metabolism [224], but they could also serve as immunomodulators to induce immunogenic death, eliminate immunosuppressive cells, and activate immune effector cells [225, 226]. In NSCLC, pemetrexed, a multi-targeted chemotherapeutic agent interfering with folate metabolic processes, strengthened the expression of HLA-G and PD-L1 by promoting glycosylation. Therefore, simultaneous targeting of PD-L1 and HLA-G could amplify the efficacy of pemetrexed in vitro [201], having implications for designing rational combination treatments for clinical trials in NSCLC. In metastatic colorectal cancer, irinotecan could be captured by HLA-G proteins, resulting in changes in pharmacokinetics and interference with the interaction between HLA-G and its receptor, but it remains ambiguous whether anti-HLA-G therapies could potentiate or attenuate the efficacy of irinotecan [140]. Most patients would suffer chemo-resistance due to the generation of cancer stem cells, while the differentiation strategies attempted to reverse the resistance by converting the rapidly proliferating malignant tumor cells to benign types [227]. However, ovarian cancer cells treated with differentiation agents showed a decrease in stemness and an increase in HLA-G and PD-L1 expression as a defense against anti-tumor immunity, indicating that the efficacy of combining differentiating drugs with chemotherapeutics might be unsatisfactory and the impact of immunosuppressive TME should be further explored [200]. On this basis, blockade of HLA-G may be a superior partner to chemotherapy for long-term effective cancer control and reversal of drug-resistant tumors.

Derangement of normal cell cycle often occurs in cancer, triggering aberrant activation of cell-cycle proteins, making targeting cyclin-dependent kinases (CDKs), or adjusting the cell cycle a beneficial approach to halt tumor growth [228]. CDK-1 is a key regulator of cell cycle progression, which interacts with Cyclin B to activate cells into M-phase and ensure normal mitosis [229]. Lovastatin synchronizes cancer cells in the G1 phase as with CDK4/6 inhibitor [230]. Nocodazole, on the other hand, prevents mitosis and induces apoptosis in tumor cells [231]. It has been shown that nocodazole and CDK1 inhibitors, but not lovastatin, could cause cell cycle blockade accompanied by increased expression of HLA-G and PD-L1, which impaired the killing effect of immune cells against tumor cells [232]. This finding may partially explain why CDK4/6 inhibitors have a better anti-tumor effect compared to other CDKs. It also suggests that blockade of HLA-G may enhance the efficacy of CDK1 inhibitors and nocodazole.

Hyperactive metabolism and disproportionate blood supplies render the tumor microenvironment hypoxic, leading to the accumulation of vascular endothelial growth factor (VEGF), which fosters angiogenesis and concomitantly arouses the upregulation of multiple immune checkpoints, including HLA-G [29, 233]. Hyperproliferation of disorganized vessels hinders immune cell infiltration to drive the development of immunosuppressive TME [234], whereas angiogenesis inhibitors exerts antitumor effects through vascular normalization and TME remodeling [235, 236]. HLA-G/ILT-4 signaling was crucial to tumor angiogenesis, documented with that the interplay between HLA-G and ILT4 boosted the expression of VEGF-C in clear cell renal cell carcinoma and NSCLC, thereby facilitating the tumor growth and lymphatic metastasis [105, 237, 238]. Thus, a combination of HLA-G blockade and angiogenesis inhibitor has been proposed, yet more compelling evidence is required to assess its efficacy.

Beyond the targeted therapies mentioned above, inhibition of AKT or ERK might coordinate with HLA-G-based therapies, as HLA-G/ILT-4 has been shown to promote tumor cell proliferation, migration, and invasion through the activation of AKT/ERK signaling in CRC [105].

Currently, therapies targeting HLA-G centered on developing specific antibodies to block the conjugation of HLA-G and its receptor, featuring the advantages of high affinity and high selectivity, yet having many shortcomings in terms of stability, immunogenicity, and production costs. In this context, orally-bioavailable small-molecule inhibitors with high drug concentration, minor rejection, and low cost are nominated as more desirable antitumor therapies [239, 240]. However, due to the complex spatial interactions between HLA-G and its ligands, competitive binding sites have not yet been well-characterized, leaving the development of small-molecule inhibitors lagging behind that of antibodies. The proteolysis-targeting chimeras (PROTACs) have revolutionized the development of small molecule drugs by harnessing the natural protein degradation system in the body to reduce protein levels [241]. Given that HLA-G could be ubiquitinated, designing suitable PROTACs linking HLA-G and E3 ligase to form a ternary complex that facilitates recognition and degradation of ubiquitinated HLA-G by the proteasome might be a novel tactic to decrease HLA-G expression. In addition to this, ablation of HLA-G using RNAi or CRISPR/Cas9 gene editing may also potentiate the anti-tumor response. In renal cell carcinoma or choriocarcinoma cells, stronger killing capacity of NK cells against tumor cells was evoked after silencing of HLA-G with the CRISPR/Cas9 system [242]. Interference with HLA-G expression using miRNA mimics diminished the viability, migration, and invasion of OSCC cells [119]. However, these two approaches are still preliminary and their application in the clinic remains to be elucidated.

Chimeric antigen receptors (CARs) are artificial receptor molecules created by genetic engineering technology, which could confer specificity to immune effector cells (e.g., T lymphocytes, NK cells) against antigenic epitope of the target, thereby reinforcing the ability of immune cells in recognizing antigenic signals and activation [243]. Nevertheless, the poor clinical efficacy of CAR-T or CAR-NK therapies may be attributed to the presence of a plethora of immunosuppressive molecules in the TME, including HLA-G. It was demonstrated that the anti-HLA-G constructs convert inhibitory signals into activating signals, initiating robust cytotoxicity of engineered CAR-NK cells upon contact with tumor cells. Low-dose chemotherapy enhanced the sensitivity of tumor cells to CAR-NK cells by raising HLA-G expression on the surface of tumor cells [22]. CAR-NK cells were characterized by higher safety, easier accessibility, and fewer side effects compared with CAR-T cells. Considering that HLA-G is limited to immune-privileged tissues and is almost undetectable in normal cells, chemotherapy-induced high expression of HLA-G in tumor cells would cause a further reduction in the damaging effects of HLA-G CAR-NK on normal tissues.

Therapies targeting HLA-G are emerging as a promising area of clinical research, with preliminary success in certain cancers. However, clinical trials with larger cohorts are needed to validate these early results and identify a safe and effective single-agent or combination modality based on HLA-G that can be widely used in clinical practice.

## Conclusions

In this review, we propose that HLA-G, as a novel IC, constructs a complex immune regulatory network by impairing or potentiating the function of key immune cells. The expression pattern of HLA-G in cancers is highly heterogeneous, with an overall trend of increasing with disease progression, suggesting that HLA-G plays an important role in the development of malignancy tumors. Hence, a growing body of preclinical work attempts to unveil novel approaches to break the HLA-G-based shield adopted by tumors to restore the anti-tumor response of immune effector cells, advancing future clinical applications. However, new means or strategies for detecting HLA-G are urgently needed given the poor consistency of existing antibodies or kits, which is of vital importance to illustrate the function of HLA-G and its different transcript isoforms. Furthermore, there are still a number of issues to be resolved in the future. By which mechanisms does HLA-G specifically regulate immune cells or tumor cells to exert immunosuppressive functions? What is the role of HLA-G signaling in the pathogenesis of oncology? What is the efficacy and safety of strategies targeting HLA-G as monotherapy or in combination with existing ICBs such as anti-PD-1/PD-L1 antibodies in the treatment of cancer? Is it feasible to develop antibodies targeting both HLA-G and ILT? How can new potential small-molecule agents be discovered to combat the limitations of monoclonal antibodies by targeting the regulatory mechanisms of HLA-G? Can sHLA-G and exosomal HLA-G be used as reliable blood-based biomarkers for early diagnosis or prediction of responsiveness to chemotherapy and ICB therapies? Despite the aforementioned issues have denoted the need for further evidence to pinpoint the intertwined role of HLA-G in cancers, HLA-G holds immense potential to be a promising biomarker for early diagnosis and prognosis assessment, and constitutes an effective targetable strategy for postponing or halting tumor growth.

## Data Availability

Not applicable.

## Abbreviations

ICB: Immune checkpoint blockade
HLA-G: Human leukocyte antigen-G
MHC: Major histocompatibility complex
NK cells: Natural killer NK cells
MDSC: Myeloid-derived suppressor cell
DNMT: DNA methyltransferase
HDACi: Histone deacetylase inhibitor
TSA: Trichostatin A
CREB: CAMP-responsive element binding protein
IRF: Interferon-regulatory factor
ISRE: Interferon-stimulated response element
HIF-1: Hypoxia-inducible factor 1
HRE: Hypoxia responsive element
HSF1: Heat shock factor 1
HSE: Heat shock element
LINE: Long interspersed nuclear elements
PRE: Progesterone response element
EGF: Epidermal growth factor
OSCC: Oral squamous cell carcinoma
CIN: Cervical intraepithelial neoplasia
GWAS: Genome-wide association
T_FH_: Follicular helper T cells
Th2: T helper 2
DCs: Dendritic cells
iNKT: Invariant natural killer T
PD-L1: Programmed death-ligand 1
NSCLC: Non-small cell lung cancer
LECs: Lymphatic endothelial cells
PBMC: Peripheral blood mononuclear cell
APCs: Antigen presenting cells
TME: Tumor microenvironment
CTLA-4: Cytotoxic T-lymphocyte-associated protein 4
TIGHT: T cell Ig and ITIM domain
TIM-3: T cell immunoglobulin domain and mucin domain-3
CDK: Cyclin-dependent kinase
VEGF: Vascular endothelial growth factor
PROTACs: Proteolysis-targeting chimeras
CARs: Chimeric antigen receptors
